# Optimization and Experiment of a Novel Compliant Focusing Mechanism for Space Remote Sensor

**DOI:** 10.3390/s20236826

**Published:** 2020-11-29

**Authors:** Yan Li, Wenjie Ge, Xu Zhang, Xinxing Tong

**Affiliations:** 1 School of Mechanical Engineering, Northwestern Polytechnical University, Xi’an 710072, China; lyan@opt.ac.cn (Y.L.); zhangxu2018@mail.nwpu.edu.cn (X.Z.); 2 Xi’an Institute of Optics and Precision Mechanics, Chinese Academy of Sciences, Xi’an 710119, China; 3 School of Mechanical and Precision Instrument Engineering, Xi’an University of Technology, Xi’an 710048, China; tongxx@xaut.edu.cn

**Keywords:** compliant mechanism, flexure amplifier, focusing mechanism, space remote sensor

## Abstract

The change of an external environment leads to the defocusing phenomenon of the space optical remote sensor. The performance of the focusing mechanism is related to the image quality of the remote sensor. It was optimized for a novel focusing mechanism comprised of a flexural hinge lever-type amplifier and several piezoelectric ceramics to improve the performance on high loads and large stroke in this research. It has advantages of a lightweight, simple structure and high reliability compared with the traditional focusing mechanism. The input displacement from the piezoelectric actuators was amplified by a two-stage flexure hinge lever-type mechanism. Dimensional parameters of the flexural hinges were considered as design variables. Based on the optimization ideology, reasonable compliance and dimension parameters of the flexural hinges were analyzed for the focusing mechanism. Simulation and experiments of deformation were conducted to validate the correctness of design optimization. The results show that the focusing mechanism designed by the proposed method has the capabilities of an amplification ratio of 100 times and a loading carrying capacity of 2 kg. This work provides a novel strategy to design an excellent focusing mechanism with lightweight, high loads and large stroke. Moreover, it is believed that this approach can be extended to other complex sensors.

## 1. Introduction

With the rapid development of the remote sensing technology, the space optical remote sensor has been widely applied in agriculture, ocean, and meteorology fields. In order to obtain a high-resolution image, the image target needs to be accurately imprinted on the sensor. Due to the impacts of vibration during the launch and the thermal radiation in orbit space, the focal plane position is defocused. The focusing mechanism is the key component of the space optical remote sensor to achieve the goal of clear image quality.

The parallel mechanism is extensively used to adjust the focal plane in the traditional method. For example, a 6 degree of freedom (DOF) parallel mechanism was designed to adjust the focal plane in the gaia space telescopes, providing a positioning accuracy of 5 microns and a resolution of 0.2 microns [1,2]. In addition, a three-dimensional high-precision focusing mechanism for the major mirror in James Webb Space Telescope was achieved via a 3 degree of freedom (DOF) parallel mechanism [3]. These traditional stiff mechanisms were actuated by motors and transmission systems such as worm and gear, ball screw, and cam. They provided high accuracy and resolution at the cost of increasing structure complexity as well as cost and reducing reliability. To address these problems in the traditional focusing mechanism, piezo actuators have been recently applied to design the focusing mechanism with higher performances [4]. The motion range of focusing mechanisms is limited because of a small stroke of the piezo actuator. Therefore, a new focusing mechanism with lightweight, higher loading capacity and a larger motion range should be designed to improve these performances.

Compliant mechanisms rely on self-deformation to achieve the purpose of transmitting the force, motion, and energy [5,6,7,8]. Therefore, it can provide a high-accuracy motion with low cost because of some impressive merits such as no clearances, no friction, and maintenance free. The compliant mechanisms are successfully utilized in many fields. The compliant mechanism opens up a further significant field for focusing mechanisms in a space remote sensor [9,10]. Xu et al. [11] designed a new type of flexible composite constant force mechanism (CCFM) based on a compliant mechanism. Wu et al. [12] investigated the degree of freedom (DOF) and motion characteristics of a compliant spherical joint and designed a novel type of six degree of freedom (DOF) compliant parallel mechanisms to provide a large load and micrometer-level positioning accuracy. Consequently, the compliant mechanism is the best candidate for the focusing mechanism with high precision. The piezoelectric actuator, which can generate large output force, high stiffness, high resolution, and fast response, is usually used to drive the ultra-precision motion and positioning [13]. However, the output displacement of the piezoelectric actuator is very small and cannot achieve the design requirement of a large displacement. Additionally, in order to achieve a large motion range for the focusing mechanism, the displacement magnifying mechanism can be employed to further increase stroke of the mechanism. Thus, a compliant mechanism is the preference for a displacement amplifier used in the focusing mechanism because it integrates merits of the compliant mechanism and amplifier together. Therefore, how to design a compliant mechanism is a worthy topic to be studied in depth.

The bridge-type and lever-type displacement amplifier are two of the most commonly encountered amplification mechanisms. For the bridge-type amplifier, the amplification ratio is independent on the structural dimension. However, the application range is restricted by its limited amplification ratio and low load capacity of bearing. Many researchers have devoted themselves into improving the displacement amplification ratio and load capacity [14,15,16,17]. As the simple calculation and flexible structure, a multi-stage lever-type amplifier has been adequately investigated and increasingly applied in the case of a large amplification ratio compared to the bridge-type amplifier. Smith et al. [18] and Lobontiu et al. [19] derived the compliance models for an elliptical hinge and corner-filleted hinge. The closed-form compliance equations were established to predict the deformation and rotation precision for the circular, parabolic, and elliptic flexural hinges [20]. Since these previous excellent works, many other models and optimization methods had been developed and applied in the design of the lever-type amplifying mechanisms [21,22]. Yong et al. [23] investigated the output characteristic of the RRR (R denotes rotating hinge) and 3-RRR micro-motion stages by analyzing the accuracies of compliance models for the flexural hinge. Ham et al. [24] designed the piezoelectric pump using a lever-type hinge mechanism. The small displacement can be amplified by 10 times through the lever-type hinge mechanism. Jung et al. [25] designed a piezoelectric drive platform, which consists of a multi-lever magnifying mechanism. Its amplification ratio is over 60 times. Choi et al. [26] presented a novel piezo driven motion stage, which employed multiple motion levers. The amplification ratio of the plane lever-type mechanism with a symmetrical structure was more than 20 times. From the above reports, the lever-type mechanism has the potential to achieve the large amplification ratio with a simple structure.

Although previously focusing mechanisms can obtain a high amplification ratio (for example, 60 times in Jung et al. [25]), the bearing capacity of these mechanisms are unsatisfactory (about a few millinewtons), which are more suitable for MEMS (micro electro mechanical system), known as minimally invasive medical devices. Therefore, both a high amplification ratio and large loading capacity are more desirable for the focusing mechanism in the space remote sensor. Thus, the method to design a novel focusing mechanism for a space remote sensor is proposed to achieve the amplification ratio of 100 times and loading capacity of 20N when compared to commercial cameras in this paper. The piezo actuator and two-stage flexural hinge lever-type mechanism are integrated in a proposed focusing mechanism. Based on the optimization theory, reasonable stiffness distribution and dimensional parameters of the circular flexural hinge are obtained for the focusing mechanism. The mechanism proposed in this paper has advantages in a compact structure and a large motion range, which makes it the best candidate as a focusing mechanism in the space remote sensor. The simulation and experimental results show that the novel focusing compliant mechanism can achieve the requirements of high loads, lightweight, and a larger motion range.

In the rest of this paper, Section 2 described the configuration of the focusing mechanism based on the two-stage flexural hinge-type lever mechanism. The design model and optimization process of the focusing mechanism are presented in Section 3. The optimization results and simulation analysis is displayed in Section 4. Section 5 gives the experimental test and evaluation of the focusing mechanism. The conclusions are drawn in Section 6.

## 2. Focusing Mechanism Based on the Two-Stage Flexural Hinge Lever-Type Mechanism

**Figure 1:**
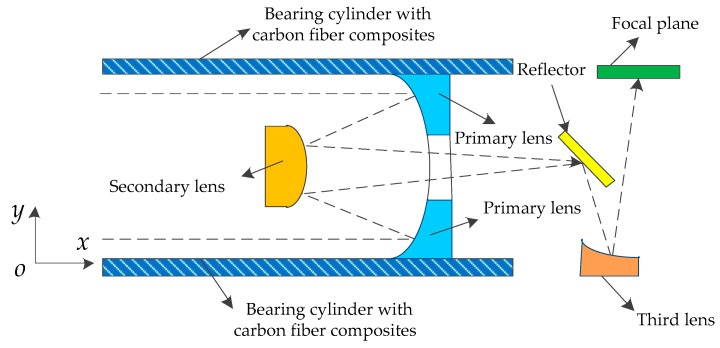
Simplified optical system schematic of a space optical remote sensor. The dashed line indicates the way of light.

The schematic of a simplified optical system in the space optical remote sensor is shown in Figure 1. The optical system is composed of four pieces of the primary lens, secondary lens, third lens, and reflector. The dimension of the telescope is 1700 mm × 650 mm × 750 mm. Its focal plane is 200 mm × 100 mm × 45 mm (length, width, and height) and the focal distance is 5 m, F/10. Focus depth is ±0.2 mm and the pixel size is 10 μm. The change of the space environment leads to the deformation of a bearing cylinder with carbon fiber reinforced resin matrix composites, which cause the out-of-focus image. According to the image-forming principle, the location of the focal plane in a space optical remote sensor should be moved along the *y* direction to obtain the clear image. Since the errors accumulation is derived from the deformation of the bearing cylinder, the focal plane needs to be moved by 2 mm to ensure a clear image. Meanwhile, the focusing mechanism also has a capability of carrying a load of 2 kg (weight of focal plane assembly). Compared with the traditional mechanism, the compliant mechanism has clear advantages and, thus, it is used to design the focusing mechanism in this research.

**Figure 2:**
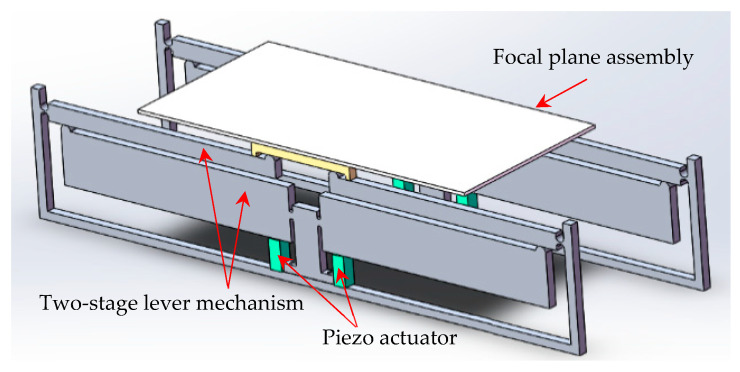
Simplified theoretically structural schematic of the focusing mechanism based on a two-stage flexural hinge lever-type mechanism. The focusing mechanism is composed of the same two sets of structural configurations.

Considering the design requirements of a space optical remote sensor, a simplified theoretically structural schematic of the focusing mechanism based on the two-stage flexural hinge lever-type mechanism is shown in Figure 2.

The interrelationships of the components are clearly shown. The focusing mechanism is composed of four sets of the same two-stage flexural hinge lever-type amplifiers. The actual focal plane assembly has an even weight distribution, which is composed of three charge coupled devices (CCD), support frames with Indium steel, printed wiring boards, and heating pipes with a weight of 2 kg. It is focused on the motion range and high load ability of the focusing mechanism in this paper. The weight is a more important point on the optimization design. Therefore, a thin rectangular volume is used to replace the actual detector. Symmetrical configuration is chosen to eliminate the parasitic displacement derived from the lever mechanism. The focusing mechanism is driven by square stack piezo actuators (PSt 150/5×5/20H, Harbin Core Tomorrow Science and Technology Co., Ltd., Harbin, China) with a maximal thrust force of 1600 N and a stroke of 20 μm. Therefore, the two-stage flexure hinge lever-type mechanism needs to be 100 times of magnification to achieve a 2-mm displacement along the vertical direction with the 2 kg weight of the detector. Materials used to manufacture the focusing mechanism are selected (with the conclusion of) the strength, weight, and cost, which will serve the satellite telescope better. Therefore, the two-stage flexural hinge lever-type mechanism is manufactured by an aluminum alloy (7075-T651) to reduce the weight and cost of the paper in which the yield strength is 503 MPa. Input displacement derived from the piezo actuator is amplified by the two-stage flexural hinge lever-type mechanism, which can push the focal plane along the vertical direction and achieve the clear image.

## 3. Optimization of the Focusing Mechanism

### 3.1. Amplification Ratio

**Figure 3:**
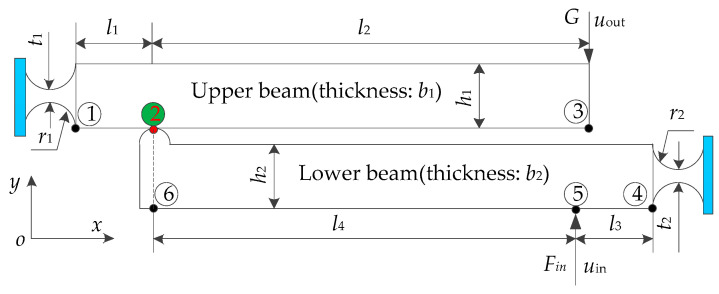
Simplified structural schematic of the two-stage flexural hinge lever-type mechanism. The upper beam and lower beam are of a mutual contact at point 2.

The two-stage flexural hinge lever-type mechanism is the key to design the amplifier. The focusing mechanism includes four forms of the same two-stage flexural hinge lever-type mechanism. To simplify the calculation model, the two-stage flexural hinge lever-type mechanism is investigated, as shown in Figure 3.

The upper beam and lower beam are assembled at point 2, which is made of aluminum alloy. Due to the symmetrical structure of the focusing mechanism, parasitic displacement in the *x* direction at the output point can be counteracted for the focusing mechanism. Therefore, we focus the displacement in the *y* direction at the output point. The symbols used in the calculation are defined in Table 1.

Deformation vectors of the upper beam are derived from the flexural hinges and constant rectangular section beam. The deformation of the symmetric circular flexural hinges at point 1 can be obtained by the Lobiotiu’s theory [27] and expressed as:

(1) $$\left[\begin{array}{l} u_{1y}^{h}\\ \theta_{1z}^{h} \end{array}\right]=\frac{12}{Eb_{1}}\int_{0}^{2r_{1}}\frac{1}{t{\left(x\right)}^{3}}\left[\begin{array}{cc} x^{2} & \;x\\ x & 1 \end{array}\right]dx\left[\begin{array}{l} F_{r}-G\\ F_{r}l_{1}-G(l_{1}+l_{2}) \end{array}\right]$$

where *E* denotes the elasticity modulus, superscript *h* indicates the displacement that comes from the flexural hinge, *F_r_* denotes the interactive force between the upper and lower beams, *r*_1_ is radius of the flexural hinge from the upper beam, *t*(*x*) is the variable thickness of a longitudinal section for the flexural hinge and is expressed as:

(2) $$t(x)=t_{1}+2\left[r_{1}-\sqrt{x(2r_{2}-x)}\right]$$

The deformation of the constant rectangular section beam can be obtained by the Euler beam theory and expressed as:

(3) $$u_{2y}^{b}=\frac{F_{r}l_{1}^{2}}{6EI_{1}}(2l_{1})+\frac{Gl_{1}^{2}}{6EI_{1}}(l_{1}-3(l_{1}+l_{2}))$$

(4) $$u_{3y}^{b}=\frac{F_{r}l_{1}^{2}}{6EI_{1}}(3(l_{1}+l_{2})-l_{1})+\frac{G{\left(l_{1}+l_{2}\right)}^{2}}{6EI_{1}}(-2(l_{1}+l_{2}))$$

where *I*_1_ denotes the inertia moment of a constant rectangular section beam around the *z* axis, while superscript *b* indicates that the displacement comes from the beam. According to the superposition theory, displacements in the *y* direction of the upper beam are expressed as:

(5) $$u_{2y}^{\mathrm{upper}}=u_{1y}^{h}+l_{1}\theta_{1z}^{h}+u_{2y}^{b}$$

(6) $$u_{\mathrm{out}}=u_{1y}^{h}+(l_{1}+l_{2})\theta_{1z}^{h}+u_{3y}^{b}$$

The deformation vector of the lower beam is similar to the upper beam. Therefore, using the same theory, displacements in the *y* direction of the lower beam can be obtained and expressed as:

(7) $$u_{\mathrm{in}}=u_{4y}^{h}+l_{3}\theta_{4z}^{h}+u_{5y}^{b}$$

(8) $$u_{6y}^{\mathrm{lower}}=u_{4y}^{h}+(l_{3}+l_{4})\theta_{4z}^{h}+u_{6y}^{b}$$

where

(9) $$\left[\begin{array}{l} u_{4y}^{h}\\ \theta_{4z}^{h} \end{array}\right]=\frac{12}{Eb_{2}}\int_{0}^{2r_{2}}\frac{1}{t{\left(x\right)}^{3}}\left[\begin{array}{cc} x^{2} & x\\ x & 1 \end{array}\right]dx\left[\begin{array}{l} F_{in}-F_{r}\\ F_{in}l_{3}-F_{r}(l_{3}+l_{4}) \end{array}\right]$$

(10) $$u_{5y}^{b}=\frac{F_{\mathrm{in}}l_{3}^{2}}{6EI_{2}}(2l_{3})+\frac{F_{r}l_{3}^{2}}{6EI_{2}}(l_{3}-3(l_{3}+l_{4}))$$

(11) $$u_{6y}^{b}=\frac{F_{in}l_{3}^{2}}{6EI_{2}}(3(l_{3}+l_{4})-l_{3})+\frac{F_{r}{\left(l_{3}+l_{4}\right)}^{2}}{6EI_{2}}(-2(l_{3}+l_{4}))$$

Due to the mutual contact between the upper and lower beam, displacements at point 2 are equivalent to the displacement at point 6. The added compatibility equation is expressed as:

(12) $$u_{4y}^{h}+(l_{3}+l_{4})\theta_{4z}^{h}+u_{6y}^{b}=u_{1y}^{h}+l_{1}\theta_{1z}^{h}+u_{2y}^{b}$$

The interactive force *F_r_* is obtained by solving Equation (12). Then, the *F_r_* is substituted into Equations (1), (3) and (4), and Equations (9)–(11). The displacements *u*_in_ and *u*_out_ in the *y* direction can be derived by Equations (7) and (6). As a result, the amplification ratio *P* of the two-stage flexural hinge lever-type mechanism is obtained and described as:

(13) $$P=\frac{u_{\mathrm{out}}}{u_{\mathrm{in}}}=\frac{u_{1y}^{h}+(l_{1}+l_{2})\theta_{1z}^{h}+u_{3y}^{b}}{u_{4y}^{h}+l_{3}\theta_{4z}^{h}+u_{5y}^{b}}$$

For completeness, all variables used in the above flexure equations are listed in Table 2.

### 3.2. Optimization Model

The dimensional parameters of the symmetric circular flexural hinge, such as the *r*_1_, *r*_2_, *t*_1_, and *t*_2_. First, determine the flexibility matrix of flexural hinges based on the Lobiotiu’s theory. Consequently, deformation along the *y* direction of the two-stage flexural hinge lever-type mechanism is severely dependent on the flexibility matrix. The dimensional parameters of the symmetric circular flexural hinge have an important effect on the amplification ratio of the focusing mechanism. In order to maximize the amplification ratio, radius and thickness of the symmetric circular flexural hinge is defined as design variables. Meanwhile, the input force of the focusing mechanism is derived from the piezo actuator. Because of the limited output displacement of the piezo actuator, allowable maximal input displacement for the flexible amplification mechanism is considered as a constraint condition. Therefore, the optimization model of the focusing mechanism is expressed as:

(14) $$\begin{array}{l} Find\;x=[r_{1},t_{1},r_{2},t_{2}]\\ \min\;P(x)=-\frac{u_{\mathrm{out}}(x)}{u_{\mathrm{in}}(x)}=-\frac{u_{1y}^{h}+(l_{1}+l_{2})\theta_{1z}^{h}+u_{3y}^{b}}{u_{4y}^{h}+l_{3}\theta_{4z}^{h}+u_{5y}^{b}}\\ s.t.\left\{ \begin{array}{l} f_{1}(x)=u_{\mathrm{in}}(x)-u_{\max}\leq 0\\ r_{\min}\leq r_{1},r_{2}\leq r_{\max}\\ t_{\min}\leq t_{1},t_{2}\leq t_{\max} \end{array}\right.\; \end{array}$$

where ***x*** is the design variable vector including the radius and thickness of flexural hinges, *u*_max_ denotes the maximal allowable input displacement, (*r*_min_, *r*_max_) are the allowable minimal and maximal radius values for a circular flexural hinge, and (*t*_min_, *t*_max_) are the allowable minimal and maximal height values for the circular flexural hinge.

### 3.3. Sensitivity Analysis and Solution

Sensitivity analysis is a key for the gradient based on the optimization algorithms to solve the optimization problem. From Equation (14), the sensitivities of objective and constraint function with respect to a change in the design variable *x* are expressed as:

(15) $$\begin{array}{ll} \frac{\partial P(x)}{\partial x} & =-\frac{\partial u_{\mathrm{out}}(x)}{\partial x}\frac{1}{u_{\mathrm{in}}(x)}+\frac{\partial u_{\mathrm{in}}(x)}{\partial x}\frac{u_{\mathrm{out}}(x)}{u_{\mathrm{in}}{\left(x\right)}^{2}}\\ & \;=-\left(\frac{\partial u_{1y}^{h}(x)}{\partial x}+\frac{\left(l_{1}+l_{2})\partial \theta_{1z}^{h}(x\right)}{\partial x}+\frac{\partial u_{3y}^{b}(x)}{\partial x}\right)\frac{1}{u_{\mathrm{in}}(x)}+\left(\frac{\partial u_{4y}^{h}(x)}{\partial x}+\frac{l_{3}\partial \theta_{4z}^{h}(x)}{\partial x}+\frac{\partial u_{5y}^{b}(x)}{\partial x}\right)\frac{u_{\mathrm{out}}(x)}{u_{\mathrm{in}}{\left(x\right)}^{2}} \end{array}$$

(16) $$\frac{\partial f_{1}(x)}{\partial x}=\frac{\partial u_{\mathrm{in}}(x)}{\partial x}=\frac{\partial u_{4y}^{h}(x)}{\partial x}+\frac{l_{3}\partial \theta_{4z}^{h}(x)}{\partial x}+\frac{\partial u_{5y}^{b}(x)}{\partial x}$$

Based on the detailed analytical Equations (1) and (4), the sensitivities in Equations (15) and (16) $\partial u_{1y}^{h}(x)/\partial x$, $\partial \theta_{1z}^{h}(x)/\partial x$, $\partial u_{3y}^{b}(x)/\partial x$ can be deduced. By deducing the analytical Equations (9) and (10), the partial derivatives $\partial u_{4y}^{h}(x)/\partial x$, $\partial \theta_{4z}^{h}(x)/\partial x$, $\partial u_{5y}^{b}(x)/\partial x$ can be obtained. Substituting these derivatives into Equations (15) and (16), sensitivities between the objective function and the constraint to design variable can be achieved.

**Figure 4:**
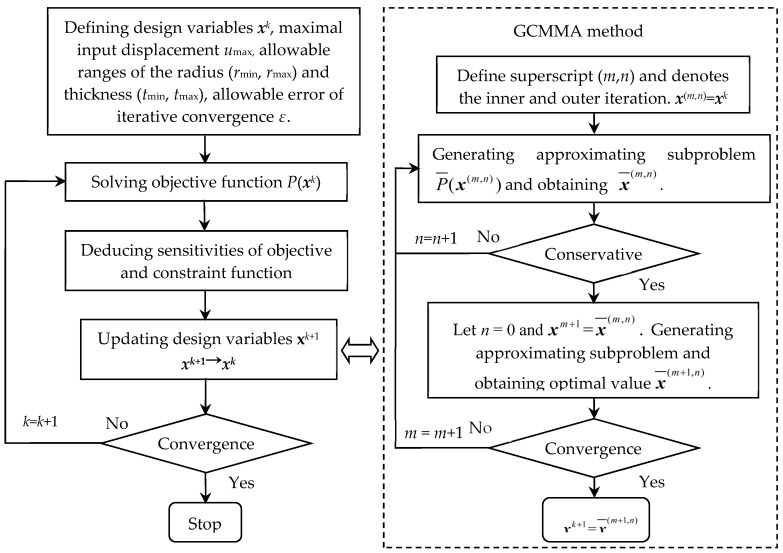
The solution flowchart of the optimization problem.

The globally convergent method of moving asymptotes (GCMMA) [28] belonging to a convex programming method is employed to solve the optimization model of the focusing mechanism. The solution flowchart of the optimization problem is shown in Figure 4. The related MATLAB codes to solve the optimization problem is provided in Appendix A. The detailed steps are as follows.

Step 1: Optimization parameters including the design variables ***x****^k^*, maximal input displacement *u*_max_, allowable ranges of the radius (*r*_min_, *r*_max_), and thickness (*t*_min_, *t*_max_) for the circular flexural hinge are first initialized. The allowable error of iterative convergence *ε* is defined and a superscript index is set as *k =* 1.

Step 2: The objective function *P*(***x****^k^*)and constraint values *f*_1_(***x****^k^*) are solved based on the current design variables ***x****^k^*.

Step 3: The sensitivity of objective and constraint functions to the change of design variable *x* are deduced based on the current design variables ***x**^k^*, respectively.

Step 4: Define superscript (*m*,*n*) and denotes the inner and outer iteration, respectively. The double index (*m*; *n*) is used to denote the *n*:th inner iteration within the *m*:th outer iteration. The allowable error of the globally convergent method of moving asymptotes (GCMMA) iterative convergence *η* is defined. Let ***x***^(*m,n*)^ = ***x****^k^*. The calculation goes into the globally convergent method of moving asymptotes (GCMMA) for the solution.

Step 5: An approximating subproblem $\bar{P}(x^{\left(m,n\right)})$ is generated at the current design variables ***x**^(m,n)^*. The optimal solution of this new subproblem is obtained by the Duality theory and denoted ${\bar{x}}^{\left(m,n\right)}$.

Step 6: If the relation of $\bar{P}({\bar{x}}^{\left(m,n\right)})\geq P({\bar{x}}^{\left(m,n\right)})$ is satisfied, the inner iteration should be at the end. Otherwise, the superscript index is updated by *n* = *n*+1 and returned to step 5.

Step 7: Let *n* = 0 and $x^{m+1}={\bar{x}}^{\left(m,n\right)}$. An approximating subproblem $\bar{P}(x^{\left(m+1,n\right)})$ is generated at the current design variables ***x**^(m^*^+1,*n*)^. The optimal solution of this new subproblem is obtained by the Duality theory and denoted as ${\bar{x}}^{\left(m+1,n\right)}$.

Step 8: If the relation of $\left|{\bar{x}}^{(m+1,n)}-{\bar{x}}^{(m,n)}\right|\leq \eta$ is satisfied, the outer iteration should be the end. Otherwise, the superscript index is updated by *m* = *m* + 1 and returned to step 5.

Step 9: The design variables vector ***x****^k^* is updated by the globally convergent method of moving asymptotes (GCMMA) and a new value is set as $x^{k+1}={\bar{x}}^{\left(m+1,n\right)}$. Based on the updated design variables ***x****^k^*^+1^, the objective function *P*(***x****^k^*^+1^) and constraint values *f*_1_(***x****^k^*^+1^) can be obtained.

Step 1: If the relation of $\left|x^{k+1}-x^{k}\right|\leq \varepsilon$ is satisfied, the optimization loop should be at the end. The optimal dimensional parameters of the circular flexural hinge can be obtained. Otherwise, the superscript index is updated by *k = k +* 1 and returned to step 2.

## 4. Simulation Results and Analysis

In order to investigate the effectiveness of the proposed method, the optimization process and results are discussed under the different input forces *F*_in_ = 600 N, *F*_in_ = 800 N, and *F*_in_ = 850 N. The initial design variable values are defined as: *r*_1_ = *r*_2_ *= t*_1_ = *t*_2_ = 1 mm. The maximal allowable input displacement derived from the piezo actuator is *u*_max_ = 0.02 mm. In order to reduce the stress values at the flexible hinges, reasonable allowable minimum values for the parameters *r*_min_, *t*_min_ are defined as 1-mm based on the repeated trial and error in a previous study. Therefore, the allowable range of the radius for a circular flexural hinge is defined as (*r*_min_ = 1 mm, *r*_max_ = 15 mm). Allowable minimal and maximal height values for a circular flexural hinge are set as *t*_min_ = 1 mm, *t*_max_ = 10 mm. The allowable error of iterative convergence is considered as *ε* = 0.001. Other structural sizes of the two-stage flexural hinge lever-type mechanism are constant parameters in the optimization process and are listed in Table 3.

**Figure 5:**
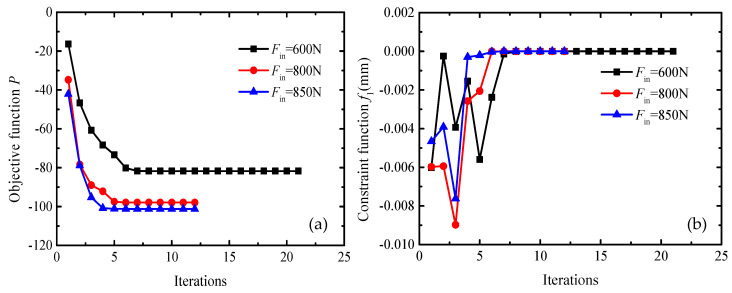
Iteration curves of the optimization process under a different input force. (**a**) The iteration curves of the optimization objective function *P*. (**b**) The iteration curves of a constraint function *f*_1_.

Figure 5 shows the iteration curves of the optimization process under different input forces.

It can be seen from Figure 5a that the three curves of the amplification ratio under input forces *F*_in_ = 600 N, *F*_in_ = 800 N, and *F*_in_ = 850 N gradually stabilize and the corresponding stable values are 81, 97, and 101, respectively. Meanwhile, the displacements at the input point under different cases also satisfy the constraint condition from Figure 5b. Therefore, the proposed method is stable convergence for the optimization problem. As the input force increases, the amplification ratio is gradually increasing. When the input force *F*_in_ is set as 600 N in order to maximize the amplification factor, the size of the flexural hinge needs to be reduced to decrease the energy lost, which overcomes the hinge deformation. This leads to the lower overall stiffness of the focusing mechanism, which is difficult to support the external load. As a result, the amplification factor of the focusing mechanism is less than the desired amplification factor of 100 times. When the input force *F*_in_ is set as 850 N, the two-stage flexural hinge lever-type mechanism can achieve the 2-mm displacement with a 5 N load. Optimal parameters of the symmetric circular flexural hinges under the input force *F*_in_ = 850 N are obtained as shown: *r*_1_ = 4.08 mm, *t*_1_ = 1 mm, *r*_2_ = 1 mm, *t*_2_ = 2.54 mm. The change of the input force leads to the change of the boundary conditions in an optimization process. The deformation and transmission ratio of the focusing mechanism are influenced by different optimal hinge sizes obtained under these boundary conditions. When the hinge sizes are given, the magnification ratio is determined. In other words, the magnification ratio does not change with the change of input force for a particular focusing mechanism.

**Figure 6:**
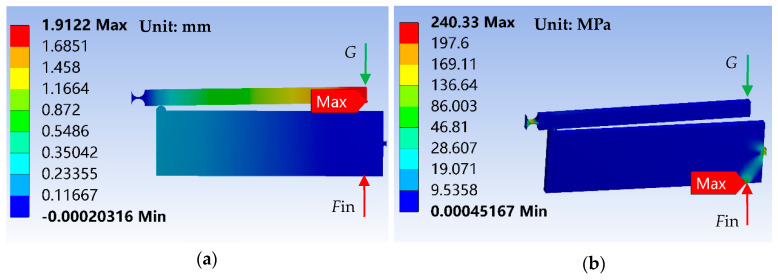
Deformation in the *y* direction and Von-Mises stress levels of a two-stage flexural hinge lever-type mechanisms based on optimal parameters under input force *F*_in_ = 850 N. (**a**) The deformation in the *y* direction of two-stage lever mechanisms, and (**b**) the Von-Mises stress levels of two-stage lever mechanisms.

According to the optimal parameters, a three-dimensional analysis model of the two-stage flexural hinge lever-type mechanism is built. The focal assembly weight is considered as concentrated force G and is applied at point 3. While the piezo actuator is allocated at point 2 and provide the input concentrated force *F*_in_. The deformation in the *y* direction and stress levels are investigated by the Finite Element Analysis (FEA), as shown in Figure 6. The input and output displacements, respectively, reach at 0.019 mm and 1.91 mm. The maximal stress is 240.29 MPa and satisfies the material’s yield strength. The simulation results are in accordance with theoretical optimization results, which illustrates the correctness and effectiveness of the proposed method.

To further reduce the weight of the focusing mechanism, optimization problems of the flexural hinges under different heights *h*_2_ = 35 mm, *h*_2_ = 32 mm of a lower beam are implemented. The input forces are increased into *F*_in_ = 950 N and *F*_in_ = 1050 N, respectively. Other constant parameters are listed in Table 3. As listed in Table 4, the optimal parameters of the symmetric circular flexural hinges under different cases are obtained by using the proposed method.

The results show that reducing the height of the lower beam can satisfy the design requirements at the expense of a larger input force. Meanwhile, due to the increase of input force, the optimal parameter *t*_2_ of the flexural hinge in the lower beam is gradually increased, which can guarantee a constraint boundary condition of the input displacement.

**Figure 7:**
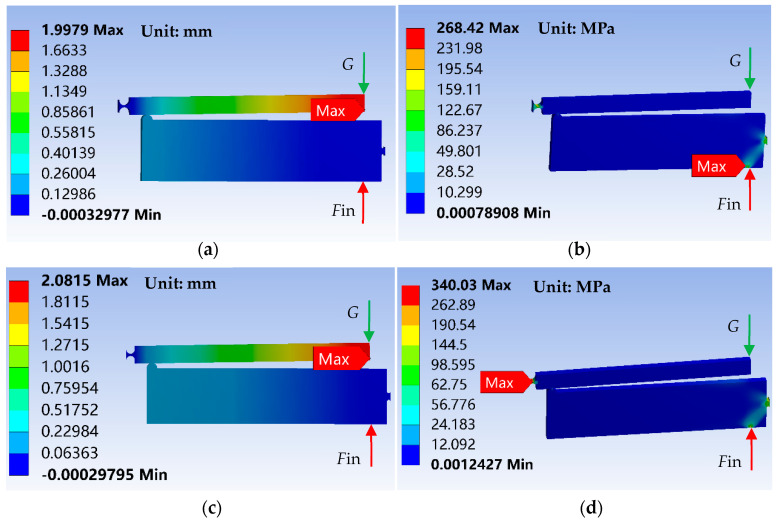
Deformation and Von-Mises stress levels of a two-stage flexural hinge lever-type mechanism under the two cases. (**a**) The displacement in the *y* direction for case 1, (**b**) the Von-Mises stress levels for case 1, (**c**) the displacement in the *y* direction for case 2, and (**d**) the Von-Mises stress levels for case 2.

Based on the above optimal parameters, three-dimensional analysis models of the two-stage flexural hinge lever-type mechanism under different cases are built. The deformation and stress levels are investigated by the FEA and are shown in Figure 7.

The input and output displacements, respectively, reach at 0.02 mm, 1.99 mm for case 1, and 0.02 mm, 2.08 mm for case 2. Maximal stress for the different cases is 268 MPa for case 1 and 340 MPa for case 2. Maximum stress happens when piezo touch the structure for case 1. Stress values in flexible hinges reach 238 MPa and a smaller value than the value of the contact area. The maximal stress values are less than the material yield strength. The results show that reduction in weight of the focusing mechanism is at the cost of the increasing stress. The simulation results correspond with theoretical optimization results. Although the weight can be reduced by increasing the input force, the yield stress of the material limits the maximum input force. After a repeated calculation, when the input force increases to 1320 N, the Von-Mises stress is 531.28 MPa and it exceeds the yield limit of the aluminum alloy (7075-T651), which can be used as a reference value for the maximum input force. Therefore, in order to satisfy the design requirements and reduce the weight for the focusing mechanism, parameters in case 2 are the optimal designing scheme.

**Figure 8:**
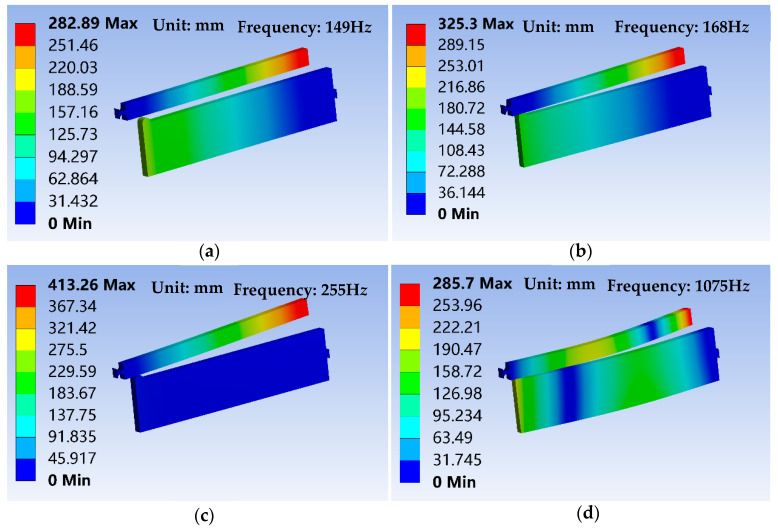
First four modes of vibration and natural frequencies of the two-stage flexural hinge lever-type mechanism. (**a**) The first order total deformation mode shape, (**b**) the second order total deformation mode shape, (**c**) the third order total deformation mode shape, and (**d**) the fourth order total deformation mode shape.

To estimate sensitivity to external vibrations, the two-stage flexural hinge lever-type mechanism using the parameters in case 2 is to consider a research objective and the modal analysis is performed to obtain natural frequency and principal mode. Figure 8 shows the first four modes of vibration and its natural frequencies of the two-stage flexural hinge lever-type mechanism. Unit (mm) in Figure 8 indicates the dimensions of the three-dimensional model and not actual deformation of the focusing mechanism.

The fundamental frequencies of the mechanism are, respectively, 149 Hz, 168 Hz, 255 Hz, and 1075 Hz, which are far larger than excitation frequency in space (generally less than 10 Hz). Thus, the mechanism designed for the space camera can successfully isolate external vibration in space, further guaranteeing image quality of the space camera.

Since the frequency of the space camera in a launch vibration is 70 Hz, which is less than the frequency of 149 Hz of focusing mechanism, there is no severe resonance phenomenon. Moreover, when the focusing mechanism is enclosed by the locking mechanism during the launch stage, the vibration has very little effect on the focusing mechanism. When working in space, the locking mechanism is opened and the focusing mechanism achieves different motion displacements by adjusting the voltage of the piezo actuator and obtain the clear image. Therefore, the focusing mechanism can meet the design requirements of the space camera.

## 5. Experiment and Evaluation

**Figure 9:**
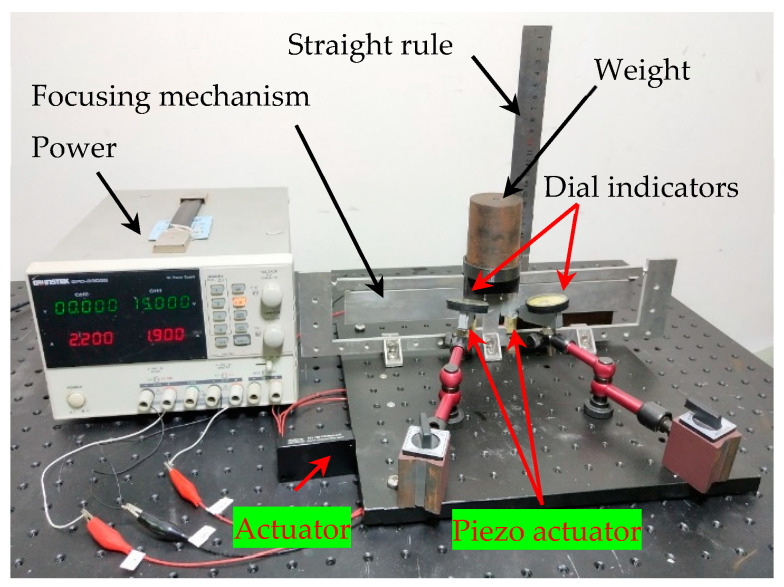
The experimental platform of the focusing mechanism based on a two-stage flexural hinge lever-type mechanism. The driven force is derived from the piezo actuator.

In order to verify the proposed method, a preliminary experiment is implemented. On the basis of the above optimal parameters of the flexural hinge, the focusing mechanism based on the two-stage flexural hinge lever-type mechanism was first manufactured by laser cutting. The experimental platform was designed and whole configuration was shown in Figure 9.

The input force was executed by the piezoelectric ceramic and the load of the focal plane was equivalently simulated with weights. The input and output displacements were, respectively, measured by the displacement dial indicators (micron-level accuracy) and the straight edge rule (millimeter accuracy). The research is mainly focused on the requirement of the focusing mechanism in the case of high load, lightweight, and a large motion range. The accuracy of the rule has slight influence on the data measured experimentally regarding the motion range. Since the output stroke of the piezo actuator was micro-scale, the lever type dial indicator was adopted to measure the displacements. It is capable of measuring up to micron accuracy. The dial indicator was fixed on the experimental platform and the measuring head was in contact with the upper surface of the piezo actuator. The input force of the focusing mechanism was derived from the piezo actuator. Working voltage of the piezo actuator was first recorded. According to the curve of voltage and output force, input force can be deduced indirectly. Output force of the focusing mechanism was equivalent to the gravity of the weight.

**Figure 10:**
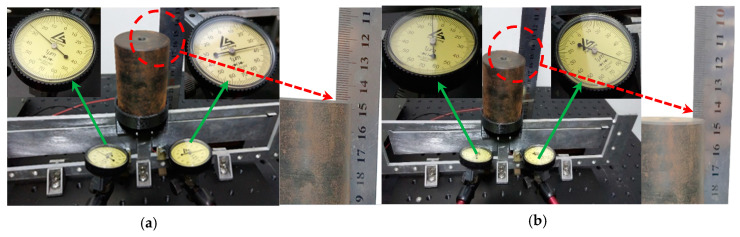
The initial and final experimental deformation of the focusing mechanism based on a two-stage flexural hinge lever-type mechanism. (**a**) Experimental deformation of the focusing mechanism with no input force and (**b**) experimental deformation of the focusing mechanism with input force.

The initial and final experimental deformation of the focusing mechanism were based on a two-stage flexural hinge lever-type mechanism, as shown in Figure 10. In the initial stage as shown in Figure 10a, the piezo actuators were not actuated by power, input force was zero, and loading weight was only actuated. Then, digits of the dial plate at input points and output points were, respectively, 27 μm, 36 μm, and 147 mm. In the final stage shown in Figure 10b, the piezo actuators were actuated by power, digits of a dial plate at input points, and output point were, respectively, 8 μm, 57 μm, and 145 mm. According to the above digits, the displacements of the piezo actuator (input point) and weight (output point) were 19 μm, 21 μm, and 2 μm. Experimental errors were derived from the processing and assembling precision, visual observation error, and measurement precision. The highly precise measurement system will be adopted to reduce the experimental error. Therefore, the focusing mechanism based on the two-stage flexural hinge lever-type mechanism approximately achieved the amplification factor of 100 times with a carrying load of 2 kg.

**Figure 11:**
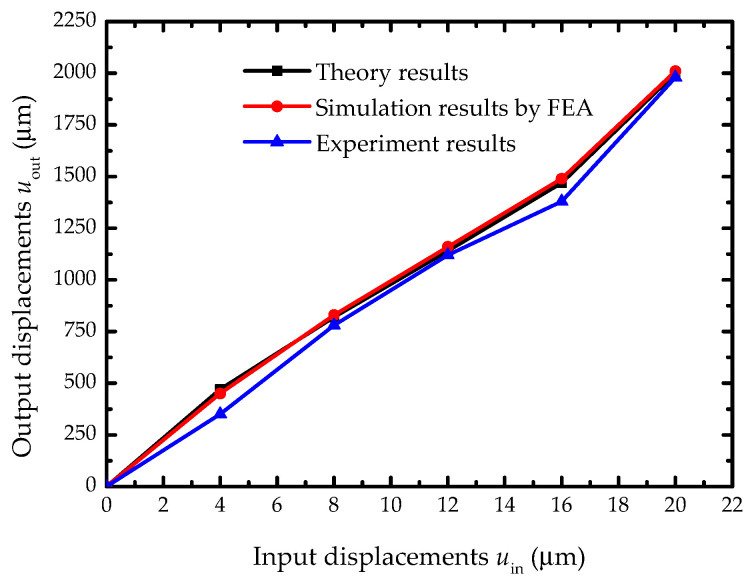
The comparison displacement curve between theory calculation, experimental deformation, and simulation results for the focusing mechanism based the two-stage flexural hinge lever-type mechanism.

In order to illustrate the amplification characteristic for the focusing mechanism, output displacements of the focusing mechanism with different input displacements were investigated by the theory calculation, FEA, and experiments. Figure 11 was the comparison displacement curves for the focusing mechanism based on the two-stage flexural hinge lever-type mechanism.

From the curves, the simulation results from FEA are in good agreement with the theory calculation. Experimental results were approximately consistent with the simulation. The relationship curve between input and output displacements is approximately linear and slopes were constant, which are approximately equal to 100. Errors analysis for the output displacement and amplifier ratio by comparing the theory, simulation, and experimental results are listed in Table 5. The errors of the output displacement *u*_out_ are equal to the amplifier ratio *P* in the simulation and experimental results.

The maximal errors of the *u*_out_ and the *P* are, respectively, 3.0% and 7.1% when the input displacement is 16 μm. Moreover, the maximal error of output displacement between the theory results and the experiment is 0.11 mm, which derives the machining precious and measurement accuracy. The focusing mechanism is manufactured by the laser cutting machine with an error range of ±0.01 mm in this paper. The output displacement is measured by the steel ruler with an error range of ±0.2 mm. According to the worst-case method and lever theory, the error range of output displacement is ±0.3 mm. Similarly, the measurement error range of the amplifier ratio is ±10 in 20 μm input displacement. Due to the focus depth of ±0.2 mm for the satellite camera, the maximum measurement error of 0.11 mm is less than the design error range. Therefore, the maximum error does not affect the clarity image and it is acceptable for focal control. The focusing mechanism proposed in the study satisfied the design requirements of the satellite camera.

In order to ensure the measurement accuracy, the contact-type dial indicator was first calibrated by the standard measuring tool before the experiment. The range of the dial indicator was 0–0.14 mm, and the division value was 0.001 mm, which satisfies the accuracy requirements. Then, the displacements of the piezo actuator under a different working voltage were measured using the calibrated contact-type dial indicator. The force-displacement curve from the measurement data was consistent with the curve obtained from the manufacturer. Therefore, this ensures the high-accuracy measurement of the input displacement derived from the piezo actuator. Since the change in the output displacement of the focal plane for the focusing mechanism was at a millimeter level, a stainless-steel rule was used to measure the displacement. Although the measures can greatly ensure the measurement accuracy in experiments, the measurement error is inevitable by using a visual observation method, which is the most important factor to generate the measurement error. In addition, the installation accuracy of the measurement system leads to the larger measurement errors. The accuracy of a contact-type dial indicator and steel ruler have a slight influence on the measurement error. A high-precision measurement system will be adopted in future research to reduce measurement errors.

## 6. Conclusions

A method to design the novel compliant focusing mechanism for a space remote sensor was proposed to achieve the high loads, light weight, and large stroke in this research. It is comprised of the flexural hinge lever-type amplifier and piezoelectric actuators. The piezo actuator was applied to provide the driving force and input displacement was amplified by a two-stage flexural hinge level-type mechanism. The reasonable circular flexural hinges for the two-stage flexural hinge level-type mechanism were designed based on an optimization ideology. The simulation and experiments were executed to verify the effectiveness of the proposed method and the conclusions are discussed below.

(1) Dimension parameters of circular flexural hinges were considered as the design variables. Maximizing the amplification ratio was defined as the optimization objective. The circular flexural hinges for a two-stage flexural hinge lever-type mechanism were optimized to satisfy the high loads, light weight, and large stroke of the focusing mechanism by the optimization strategy.

(2) With the growth of the input force, the amplification ratio of the two-stage flexural hinge lever-type mechanism increase. In order to guarantee the constraint condition of input displacement, minimal thickness of the optimal flexural hinge in a lower beam become thicker.

(3) When compared among the theory calculation, simulation, and experimental results, it was shown that the design method was correct and effective for the focusing mechanism. Moreover, the focusing mechanism designed by the proposed method is able to satisfy the design requirements and achieve the goals of the amplification ratio of 100 times and carrying load of 2 kg.

In a future study, mechanism precision, dynamical behaviors, and the precise control of the focusing mechanism will be investigated to improve the motion precision. The influences of uncertainty (friction, hysteresis) and sensitivity of material on experimental offsets will be evaluated to improve the motion precision. Moreover, the high-accuracy measurement system will be applied to evaluate the experimental motion precision of the focusing mechanism. On the other hand, the mechanism needs to apply a large input force to enhance load-bearing capacity by posing a challenge for the structural stiffness and control system.

## Figures and Tables

**Table 1:** Symbols used in the calculation modeling.

Symbol	Description
*l*_1_, *l*_2_, *l*_3_, *l*_4_	Beam lengths along the *x* direction
*r*_1_, *r*_2_	Radius of different flexural hinges
*t*_1_, *t*_2_	Thickness of a longitudinal section for the different flexural hinges
*h*_1_, *h*_2_	Height of different beams
*b*_1_, *b*_2_	Thickness of different beams
*F*_in_	Input force from the piezo actuator
*u*_in_ *, u*_out_	Input and output displacements
*G*	External load from the focal plane

**Table 2:** All variables are used in the above flexure equations.

Symbol	Description
$u_{1y}^{h}$	Displacement in the *y* direction at point 1 from hinge 1
$\theta_{1z}^{h}$	Rotation angles in the *z* axis at point 1 from hinge 1
$u_{4y}^{h}$	Displacement in the *y* direction at point 4 from hinge 2
$\theta_{4z}^{h}$	Rotation angles in the z axis at point 4 from hinge 2
$u_{2y}^{b}$	Displacement in the *y* direction at point 2 from the upper beam
$u_{3y}^{b}$	Displacement in the *y* direction at point 3 from the upper beam
$u_{5y}^{b}$	Displacement in the *y* direction at point 5 from the lower beam
$u_{6y}^{b}$	Displacement in the *y* direction at point 6 from the lower beam
*I*_1_, *I*_2_	Moment of inertia for the lower and upper beam

**Table 3:** Structural sizes of the two-stage flexural hinge lever-type mechanism.

Parameters	Values
Beam lengths *l*_1_(mm)	10
Beam lengths *l*_3_ (mm)	10
Beam lengths *l*_2_ (mm)	126
Beam lengths *l*_4_ (mm)	126
Beam thickness *b*_1_(mm)	10
Beam thickness *b*_2_ (mm)	10
Height of the upper beam *h*_1_ (mm)	10
Height of the lower beam *h*_2_ (mm)	40
Elasticity modulus *E* (MPa)	71,000
External load *G* (N)	5

**Table 4:** Optimal results and parameters of symmetric circular flexural hinges under two cases.

Parameters	Case 1	Case 2
Input force *F*_in_ (N)	950	1050
Height of lower beam *h*_2_ (mm)	35	32
Input displacement *u*_in_ (μm)	20	20
Amplification ratio *P*	100	100
Radius of flexural hinges *r*_1_ (mm)	3.20	2.44
Thickness of flexural hinges *t*_1_ (mm)	1	1
Radius of flexural hinges *r*_2_ (mm)	1	1
Thickness of flexural hinges *t*_2_ (mm)	2.91	3.56

**Table 5:** Error analysis for the output displacement and amplifier ratio by comparing the theory, simulation, and experiment results.

Input Displacement *u*_in_ (μm)	Output Displacement *u*_out_ and Error					Amplifier Ratio *P* and Error				
	Theory Value (μm)	Simulation		Experimental		Theory Value	Simulation		Experimental	
		Value (μm)	Error (%)	Value (μm)	Error (%)		Value	Error (%)	Value	Error (%)
4	410	405	1.2	383	6.5	102.5	101.25	1.2	95.8	6.5
8	813	830	2.1	780	4.0	101.6	103.75	2.1	97.5	4.0
12	1215	1242	2.2	1156	4.8	101.3	103.5	2.2	96.3	4.8
16	1548	1595	3.0	1438	7.1	96.8	99.7	3.0	90.0	7.1
20	2000	2010	0.5	1960	2.0	100	100.5	0.5	98.0	2.0

## Appendix A

%% Main program for solving the optimization problem
clear
b1=5; h1=10; L1=10; L2=126;
b2=5; h2=40; L3=10; L4=126;
E=71e3; F=850; G=5;
I1=b1*h1^3/12; I2=b2*h2^3/12;
x = 4*ones(4,1);
umin=0.02;
loop = 0;
change = 1;
% Sensitivity analysis
[dPdx,dyindx]=Sensitivity_analysis;
% Loop iteration
while change > 0.0001&&loop<500
loop = loop + 1;
xold = x;
% The solving function GCMMAupdate can be obtained by reference the Krister Svanberg.
[x,f0val,fval]=GCMMAupdate(b1,L1,L2,b2,L3,L4,I1,I2,E,F,G,umin,dPdx,dyindx,x);
change = max(abs(x-xold));
f0(loop)=f0val;
f1(loop)=fval;
fprintf(‘ It.:%5i Obj.:%7.4f fval.:%7.3f ch.:%7.3f\n ‘,loop,f0val,fval,change);
end
%% Subroutine for sensitivity analysis
function [dPdx,dyindx]=Sensitivity_analysis
syms r1 t1 b1 L1 L2 r2 t2 b2 L3 L4 E F G I1 I2
C1f=3/(4*E*b1*(2*r1+t1))*(2*(2+pi)*r1+pi*t1+8*r1^3*(44*r1^2+28*r1*t1+5*t1^2)/(t1^2*(4*r1+t1)^2)+...
((2*r1+t1)*(t1*(4*r1+t1))^(1/2)*(-80*r1^4+24*r1^3*t1+8*(3+2*pi)*r1^2*t1^2+4*(1+2*pi)*r1*t1^3+pi*t1^4)-...
8*(2*r1+t1)^4*(-6*r1^2+4*r1*t1+t1^2))*atan((1+4*r1/t1)^(1/2))/(t1^5*(4*r1+t1)^5)^(1/2));
C1m=24*r1^2/(E*b1*t1^3*(2*r1+t1)*(4*r1+t1)^3)*(t1*(4*r1+t1)*(6*r1^2+4*r1*t1+t1^2)+…
  6*r1*(2*r1+t1)^2*(t1*(4*r1+t1))^(1/2)*atan((1+4*r1/t1)^(1/2)));
Theta1m=C1m/r1;
C2f=3/(4*E*b2*(2*r2+t2))*(2*(2+pi)*r2+pi*t2+8*r2^3*(44*r2^2+28*r2*t2+5*t2^2)/(t2^2*(4*r2+t2)^2)+...
((2*r2+t2)*(t2*(4*r2+t2))^(1/2)*(-80*r2^4+24*r2^3*t2+8*(3+2*pi)*r2^2*t2^2+4*(1+2*pi)*r2*t2^3+pi*t2^4)-...
8*(2*r2+t2)^4*(-6*r2^2+4*r2*t2+t2^2))*atan((1+4*r2/t2)^(1/2))/(t2^5*(4*r2+t2)^5)^(1/2));
C2m=24*r2^2/(E*b2*t2^3*(2*r2+t2)*(4*r2+t2)^3)*(t2*(4*r2+t2)*(6*r2^2+4*r2*t2+t2^2)+…
  6*r2*(2*r2+t2)^2*(t2*(4*r2+t2))^(1/2)*atan((1+4*r2/t2)^(1/2)));
Theta2m=C2m/r2;
Fr =((C2m*F + F*L3*Theta2m)*(L3 + L4) + L1*(C1m*G + G*Theta1m*(L1 + L2)) + C2f*F + C1f*G+…
  C2m*F*L3 + C1m*G*(L1 + L2) + (F*L3^2*(2*L3 + 3*L4))/(6*E*I2) + (G*L1^2*(2*L1 + 3*L2))/(6*E*I1))/(C1f +…
  C2f + L1*(C1m + L1*Theta1m) + C2m*(L3 + L4) + (C2m + Theta2m*(L3 + L4))*(L3 + L4) + C1m*L1 +…
  (L3 + L4)^3/(3*E*I2) + L1^3/(3*E*I1));
w1=C1f*(Fr-G)+C1m*(Fr*L1-G*(L1+L2));
theta1=C1m*(Fr-G)+Theta1m*(Fr*L1-G*(L1+L2));
w3=Fr*L1^2*(3*(L1+L2)-L1)/(6*E*I1)-G*(L1+L2)^3/(3*E*I1);
w4=C2f*(F-Fr)+C2m*(F*L3-Fr*(L3+L4));
theta4=C2m*(F-Fr)+Theta2m*(F*L3-Fr*(L3+L4));
w5=F*L3^3/(3*E*I2)-Fr*L3^2*(3*(L3+L4)-L3)/(6*E*I2);
yin=w4+L3*theta4+w5;
yout=w1+(L1+L2)*theta1+w3;
P=-yout/yin;
dPdr1=diff(P,r1); dPdt1=diff(P,t1);
dPdr2=diff(P,r2); dPdt2=diff(P,t2);
dyindr1=diff(yin,r1); dyindt1=diff(yin,t1);
dyindr2=diff(yin,r2); dyindt2=diff(yin,t2);
dPdx=[dPdr1;dPdt1;dPdr2;dPdt2];
dyindx=[dyindr1;dyindt1;dyindr2;dyindt2];
end
